# Melanoma Cellular Plasticity

**DOI:** 10.3390/ijms23126401

**Published:** 2022-06-08

**Authors:** Daniel Novak, Jochen Utikal

**Affiliations:** 1 Skin Cancer Unit, German Cancer Research Center (DKFZ), 69121 Heidelberg, Germany; d.novak@dkfz-heidelberg.de; 2 Department of Dermatology, Venereology and Allergology, University Medical Center Mannheim, Ruprecht-Karl University of Heidelberg, 68167 Mannheim, Germany; 3 DKFZ Hector Cancer Institute, University Medical Center Mannheim, 68167 Mannheim, Germany

Despite the advances of modern medicine and the development of innovative and promising new therapeutic strategies for the treatment of the numerous types of cancer, far too many patients still lose the battle against these devastating diseases. This is due to, among other things, the phenotypic heterogeneity of cancer cells that goes along with different physiological and functional properties. Two cancer cells from the same tumor can therefore respond quite differently to a certain type of treatment and, in the worst case, not respond at all. Malignant melanoma is an exceptionally aggressive type of skin cancer whose cells exhibit a high grade of phenotypic heterogeneity. This Special Issue of the *International Journal of Molecular Sciences* addresses the cellular plasticity of melanoma and its consequences for therapy.

Cellular plasticity and heterogeneity can find expression in a variety of molecular, physiological and functional characteristics. An obvious sign of plasticity among melanocytes and melanoma cells is the level of pigmentation that can vary to a considerable degree. As reviewed recently by Slominski and colleagues, a higher pigmentation of a melanoma tumor negatively correlates with patient prognosis. Moreover, there is evidence that melanin not only protects cells from harmful UV light but might also be relevant for melanomagenesis. Intermediate products of melanin synthesis have been found to exhibit mutagenic, cytotoxic and immune suppressive features. Additionally, melanin can also interfere with radio- and chemotherapy and in this way reduce the success of these established therapy methods [1]. This example and the following research publications and reviews featured in this Special Issue clearly show that cellular plasticity significantly affects the course of melanoma pathogenesis and therapy. 

Hakobyan and colleagues [2] analyzed and compared the transcriptomes of benign melanocytic nevi and primary melanomas in order to identify splice variants of genes whose expression differs between melanoma cells and melanocytes. In this analysis, a great number of genes was found to show differential isoform expression, with RAB6B, MSR1, COLL11A2 and LYPD1 exhibiting the highest difference. Interestingly, mutations of genes that are important for splicing processes were found in melanocytes from benign nevi and melanoma cells. However, significantly more mutations in genes of the splicing machinery were present in the melanoma cells. These data indicate that aberrations in genes relevant for splicing occur at an early stage of melanomagenesis and might account for the differential expression of isoforms between melanocytes and melanoma cells.

An important feature of cancer cells is their ability to affect their environment in a way that enables growth and metastasis. This is often achieved by the secretion of specific factors. In this context, Anchan and colleagues report in this Special Issue that melanoma cells perturb the integrity of the endothelial barrier in the brain. Although the secretomes of distinct melanoma cell lines differ more or less, they all exert a comparable effect. Interestingly, it seems that not a single factor but the concerted action of a variety of factors is responsible for the altered brain endothelial barrier integrity [3].

Plasticity among melanoma specimens is one of the reasons that some patients respond to a certain treatment while others do not. Beberok and colleagues [4] examined the cytotoxicity of the synthetic antibiotic lomefloxacin in combination with UVA radiation towards amelanotic and melanotic melanoma cells. They could demonstrate that the combined application of lomefloxacin and UVA radiation is much more effective in reducing the cell viability of melanoma cells than the application of the antibiotic alone. Moreover, the amelanotic as well as the melanotic melanoma cells respond equally to the treatment suggesting a potential broader applicability for melanoma therapy.

Combinatorial treatment is a promising approach to overcome resistance issues and to enhance the efficacy of established therapeutic drugs. Da-Costa-Rocha and Prieto [5], in their latest study, investigated the antiproliferative effect of selective inhibitors of cyclooxygenase (COX) and lipoxygenase (LOX) enzymes on B16F10 melanoma cells and discovered that LOX inhibitors exert more cytotoxicity compared to COX inhibitors. They also combined these drugs with the established inhibitors dacarbazine and temozolomide showing that only one COX inhibitor enhances their efficacy while LOX inhibitors antagonize them.

Apart from these highly informative research publications, this Special Issue of the *International Journal of Molecular Sciences* also features reviews that sum up the latest knowledge on a variety of topics related to cellular plasticity of melanoma. Wessely and colleagues give an overview of the neural crest (NC) origin of the melanocytic lineage and summarize how that ancestry and the high cellular plasticity of melanoma influence the course of melanoma pathogenesis with a focus on the development of resistances to current established therapies. In this context, they also shine a light on the role of NC-associated transcription factors on melanomagenesis [6]. Plasticity within a melanoma tumor represents a big challenge for current therapeutic efforts. Interestingly, the tumor microenvironment (TEM) is also subjected to changes and cells of the TEM and the tumor affect each other reciprocally. The impact of the TEM on melanoma progression, the development of resistances and immune evasion is neatly summarized in two reviews by Simiczyjew and colleagues, as well as by Romano and colleagues, in this Special Issue [7,8]. Savoia and colleagues give a comprehensive overview of the use of BRAF inhibitors for the treatment of advanced melanoma, discussing their benefits as well as the development of resistances and how to possibly overcome this hurdle [9]. Lastly, Granados and colleagues outline the latest facts about complete and partial reprogramming of cancer cells into induced pluripotent cancer (iPC) cells as a model to mimic and study cellular plasticity of melanoma in vitro [10].

Overall, this Special Issue of the *International Journal of Molecular Sciences* combines informative reviews and up-to-date research articles that emphasize the importance of investigating and understanding the mechanisms behind cellular plasticity of melanoma in order to combat this devastating disease. 
